# Route of Arsenic Exposure Differentially Impacts the Expression of Genes Involved in Gut-Mucosa-Associated Immune Responses and Gastrointestinal Permeability

**DOI:** 10.3390/ijms24076352

**Published:** 2023-03-28

**Authors:** Kuppan Gokulan, Aakriti Mathur, Amit Kumar, Michelle M. Vanlandingham, Sangeeta Khare

**Affiliations:** 1 Division of Microbiology, National Center for Toxicological Research, US Food and Drug Administration, 3900 NCTR Rd, Jefferson, AR 72079, USA; 2 Division of Biochemical Toxicology, National Center for Toxicological Research, US Food and Drug Administration, 3900 NCTR Rd, Jefferson, AR 72079, USA

**Keywords:** arsenic, intravenous, exposure route, immunity, intestine, mucosa, oral, permeability

## Abstract

First-pass metabolism alters arsenic biotransformation and its immunomodulatory activities. This study aims to determine the mRNA expression of intestinal-immunity- and permeability-associated genes, levels of cytokine/chemokines and levels of immunoglobulin isotypes when CD-1 mice were exposed to a single dose of intravenous (IV) sodium arsenite (50 µg/kg body weight (BW)) and to compare these responses to exposure via oral gavage (OG) (50 µg/kg BW). Samples were collected at 1, 4, 24 and 48 h post IV exposure and 24 and 48 h post OG. Sodium arsenite IV exposure led to a transient modulation of mRNA expression and protein levels of immunity-related genes involved in inflammation/apoptotic pathways and production of cytokines/chemokines, whereas it also led to downregulated expression of genes encoding tight junction, focal adhesion, and gap junction proteins, which are responsible for maintaining cell permeability. Oral exposure perturbed fewer cell-permeability-related genes at 24 and 48 h post exposure. At 24 h post exposure, OG decreased IgA and IgG2b levels; however, IV exposure significantly increased IgG2b, IgG3 and IgA in ileal tissue. Earlier, we showed significant downregulation of mRNA expression of genes involved in the immune-related pathways during OG in the intestinal mucosa of the same animals. Cumulatively, these results provide evidence that the exposure route of a xenobiotic can differentially impact the intestinal responses due to the impact of first-pass metabolism.

## 1. Introduction

Arsenic exposure can lead to the development of severe forms of skin, lung, bladder, kidney, and liver cancer [1]. Currently, it is noted that about 140 million people in 50 countries have been drinking water containing arsenic at levels above the WHO provisional guideline value of 10 μg/L [1,2]. Long-term arsenic exposure is known to cause arsenicosis, which can lead to gangrene and keratosis that cause major tissue death [2]. Arsenic exists in both organic and inorganic forms in nature. The toxicity of these different forms is dependent on its chemical composition and valency [3]. Inorganic arsenic exists in two forms: arsenate (As 5+) and arsenite (As 3+). At the molecular level, the toxicity of the species is caused by its interaction with thiol-containing residues of peptides and proteins. The induction of oxidative reactive species causes oxidative stress, while inhibiting antioxidant defense mechanisms, leading to cell death [3]. Such toxic forms of arsenic are abundant in nature. Moreover, a principal environmental concern is increasing arsenic contamination in groundwater sources [4]. The use of contaminated water increases the susceptibility to several autoimmune-related diseases and cancers [5,6,7]. The US National Research Council has noted that as many as 1 in 100 additional cancer deaths could be expected from a lifetime exposure to arsenic-contaminated drinking water containing 50 μg/mL [2]. In most cases, arsenic can enter the body either orally from ingestion of contaminated products, through intravenous (IV) medical treatments or during prenatal development because of parental exposure [8,9,10,11,12,13,14]. The main difference between oral and intravenous routes of exposure is how arsenic is metabolized [15,16]. Via the oral route, arsenic travels through the gastrointestinal tract and undergoes first-pass metabolism. During this phase, arsenic interacts with intestinal metabolic enzymes and gut microbiota, which metabolize it into its other toxic form. This is as opposed to IV arsenic exposure, which by-passes this first-pass metabolism [15,16,17]. In another study, oral exposure to arsenic in mice exhibited Th1 immune response coupled with IgA class switching geared towards modifying the immune system for increased risk of infections and chronic diseases such as cancer [18]. 

While research has had an intense focus on the effects of arsenic exposure on specific cancers, there is limited information on the role of acute arsenic exposure in maintaining gastrointestinal health. Continuous or repeated exposure to arsenite leads to an increase in the abundance of bacteria with arsenic resistance genes and a subsequent, transient decrease in the recovery of commensal intestinal bacteria [14,19,20,21,22]. However, the focus of our research is to study the effect of single short-term exposure on intestinal homeostasis with an emphasis on the intestinal epithelial layer permeability and gut-mucosa-associated immune responses [15,16,22,23]. Previous studies from our laboratory have shown that low levels of arsenic exposure via oral gavage (OG) can cause an imbalance in gut homeostasis even after a single exposure [22,23]. Sodium arsenite exposure can disturb gastrointestinal homeostasis by changing the bacterial community structure of gut microbiota. In the same study, sodium arsenite exposure has also been found to perturb expression of immune-related and inflammatory signaling pathways, such as nuclear factor κB (NFκB), extracellular signal-regulated protein kinases 1 and 2 (ERK1/2), p38 and myeloid differentiation protein-88 (Myd88) in the gastrointestinal tract [23]. Overall, the mRNA expression of apoptosis, inflammasomes and inflammatory response genes were significantly downregulated in the animals exposed to sodium arsenite. 

The intestinal epithelial layer acts as a barrier to separate the lumen and other mucosal tissues. The mucosa acts as a barrier with selective permeability, allowing the passage of nutrients, electrolytes, and water, while also protecting against toxins and pathogens [24]. Specifically, the tight junctions found in the paracellular space regulate the flow of molecules across the cellular barrier. Impairment of barrier function is considered a critical determinant in the predisposition to inflammatory processes causing intestinal autoimmune diseases including Crohn’s disease, ulcerative colitis, food allergies and functional disorders of the intestine such as irritable bowel syndrome [25,26]. It is evident that during short-term oral exposure, sodium arsenite undergoes metabolism in the gastrointestinal tract [15,16]. However, there is limited evidence exploring the effect of sodium arsenite via IV exposure to the intestinal epithelial cell and its ability to regulate gut permeability. The consequences of arsenic exposure to epithelial cells are of health concern, as there is evidence of a correlation between intestinal permeability and the function of the mucosal immune system. Of utmost concern is the exposure of infants and young children, who, if exposed during prenatal and critical developmental stages, can experience severe developmental complications in later stages of life. A study conducted by Kile et al. found that prenatal exposure to arsenic was associated with increased risk of health complications in adulthood [8,27]. The cohort of mothers who participated in this study showed a shortened gestation period, while their infants experienced significant decreased birth weight. The consequences of IV exposures are severe enough to demonstrate the importance of studying this route of arsenic exposure. A study by our group has also shown that the IV injection of arsenite resulted in the accumulation of arsenic species in the intestinal tissue [15,16].

First-pass metabolism has tremendous effects on arsenic metabolism before reaching systemic circulation in the body. The central hypothesis of this study is that the exposure route (oral vs. intravenous) of sodium arsenite impacts the gut mucosa responses likely due to the first-pass metabolism. Intravenously exposed arsenite reaches the gastrointestinal tract via mesenteric arteries that carry blood from the aorta and distribute it to a large portion of the gastrointestinal tract. Moreover, when arsenic is exposed orally, it interacts with intestinal mucosal immunity directly. Thus, IgA would be relevant to study, as it is highly concentrated in the intestinal tissues. In contrast, since arsenic is brought to the gastrointestinal tract via the mesenteric arteries, IgG and IgM would be relevant in the context of assessing the effects of IV route of exposure. The immediate goal of this study is to address the knowledge gap regarding how different routes of exposure can affect intestine-related immune responses as well as intestinal mucosal layer permeability. This study aims to contribute to the understanding of gut-related effects of arsenic exposure via different routes of exposure (OG vs. IV exposure) in the mouse model.

## 2. Results

Exposure of sodium arsenite via OG or IV exposure did not produce any changes in the immediate health of animals, as assessed for 48 h. As we expected that the IV exposure would lead to rapid systemic circulation of sodium arsenite and would change the intestinal-mucosa-associated responses, animals were also sacrificed at 1 and 4 h post exposure for the IV experimental group.

### 2.1. Route of Exposure (Oral or Intravenous) of Sodium Arsenite Perturbs Different Functional Pathways in the Ileal Mucosa

Gastrointestinal homeostasis is maintained by the balance in the barrier function of the intestinal epithelial cells and gut-mucosa-associated immune responses. We assessed the mRNA expression of genes involved in the perturbation of these two pathways: immune response and epithelial cell–cell junction. As shown in Table 1, the IV exposure modulated transient expression of mRNA genes in the immune-response-related pathway. In contrast, IV exposure of sodium arsenite perturbed the mRNA gene expression involved in the epithelial cell junction integrity more severely, as compared to sodium arsenite exposure via oral gavage (Table 2).

### 2.2. Exposure to Sodium Arsenite via IV Route Results in a Transient Modulation of mRNA Expression of Genes Involved in the Gut-Mucosa-Associated Immune Response

**Figure 1:**
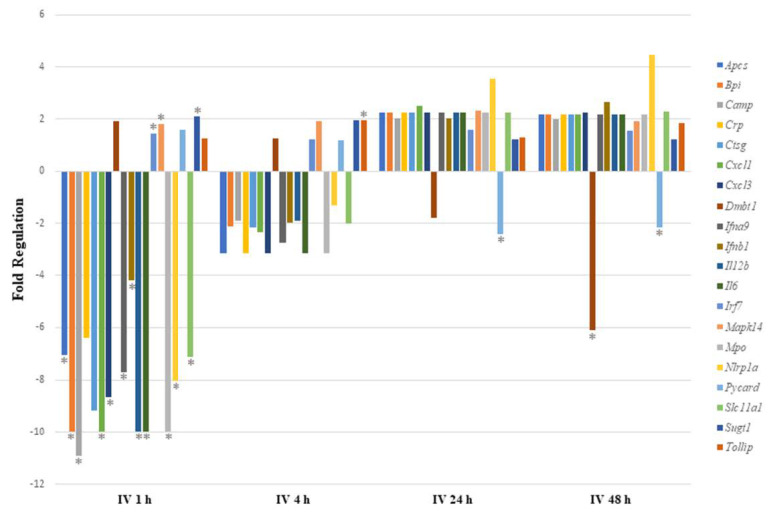
**Comparing immune response pathway genes with significant regulation after intravenous exposure to sodium arsenite.** This plot shows the fold regulation of significantly regulated gut-associated immune-response-related genes in ileal tissue of mice exposed to arsenic for 1 h, 4 h, 24 h and 48 h. Significance is represented as a star, where significance indicates *p* ≤ 0.05; *p*-values were calculated based on a Student’s *t*-test of the replicate 2^(−ΔΔCT) values for each gene in the control group and treatment groups.

We expected that IV exposure would lead to rapid systemic circulation of sodium arsenite. Thus, gene expression analysis was also conducted for 1 and 4 h post IV exposure. As shown in Figure 1, sodium arsenite exposure resulted in the differential regulation of the mRNA expression of several genes involved in gut-mucosa-associated immune responses. However, this perturbation was transient. Genes were most significantly differentially expressed at 1 h after IV exposure to arsenite, as shown in Figure 1.

**Figure 2:**
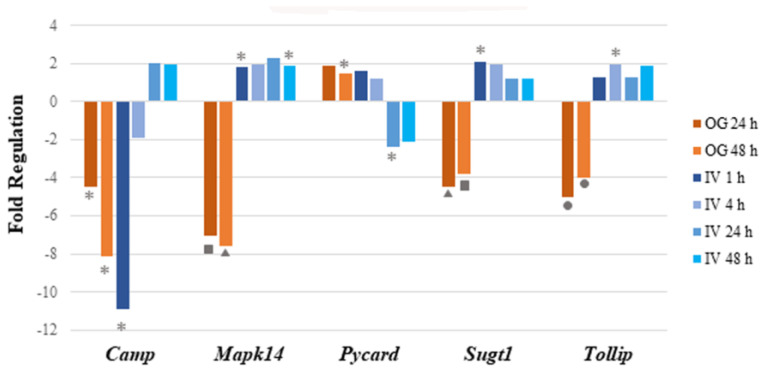
**Perturbation of mRNA expression of immune pathway genes that are common in both IV and OG exposure routes.** Bar graph shows the fold regulation of differentially expressed genes after both IV and OG exposure. The genes that were commonly regulated in both treatment groups showed no notable pattern in terms of common immune-related functions or involved pathways. Significance is indicated by the star, triangle, square and circle shapes. The legend below the plot corresponds the symbols for significance with a range of *p* values; *p* values (* = p<0.05; ▲ = p<0.01; ■ p<0.001; ● p<0.0001) were calculated based on a Student’s *t*-test of the replicate 2^(−ΔΔCT) values for each gene in the control group and treatment groups.

Of the 17 genes significantly regulated, 14 were downregulated and 3 (*Irf7*, *Mapk14* and *Sugt1*) were upregulated at 1 h post arsenite exposure. Among the 14 downregulated genes, 6 were related to inflammatory responses (*Apcs*, *Crp*, *Cxcl1*, *Cxcl3*, *Il6,* and *Slc11a1*), 7 encoded cytokines or chemokines (*Cxcl1*, *Cxcl3*, *Ifna9*, *Ifnb1, Il12b*, *Il6,* and *Slc11a1*), and 6 genes encode antimicrobial peptides (*Apcs*, *Bpi*, *Camp*, *Crp*, *Ctsg,* and *Mpo*). At 4 h after IV sodium arsenite exposure, several genes were perturbed, however, only *Tollip* was significantly upregulated. At 24 h after IV exposure, only *Pycard* was significantly downregulated. At 48 h after IV exposure, *Dmbt1* and *Pycard* were significantly downregulated. Route of exposure of sodium arsenite, in some cases, resulted in the activation of the same gene; however, the perturbation of the genes tended to be in the opposite direction, as observed in Figure 2.

In conclusion, sodium arsenite exposure via the IV route showed a transient modulation of immune-response-related genes at 1 h post exposure. To our surprise, these results were very different when the route of exposure was OG, where downregulation of genes involved in the gut-mucosa-associated immune response was noticed [23]. A detailed comparison of these modes of exposure is provided in the discussion.

### 2.3. Exposure to Sodium Arsenite via IV Route Results in the Higher Perturbation of Genes Involved in the Intestinal Permeability When Compared to OG Route

**Figure 3:**
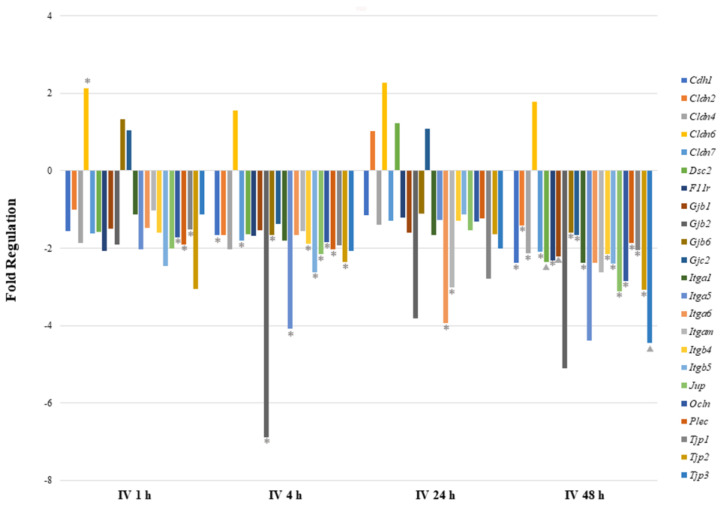
**Comparison of mRNA expression of cell junction pathway genes after intravenous exposure to sodium arsenite.** This graph shows the fold regulation of significantly regulated cell junction genes in ileal tissue of mice exposed to arsenic intravenously (IV) for 1, 4, 24 and 48 h. Significance is represented as a star or a triangle. The legend shown below the plot indicates the range of *p*-values represented by either marker. The *p*-values (* = p<0.05; ▲ = p<0.01) were calculated based on a Student’s *t*-test of the replicate 2^(−ΔΔCT) values for each gene in the control group and treatment groups.

As shown in Table 2 and Figure 3, cell–cell junction genes were most significantly perturbed after 48 h of IV exposure. Most of the downregulated genes encode tight junction proteins (*Cldn4*, *Cldn7*, *F11r*, *Ocln*, *Tjp1*, *Tjp2*, and *Tjp3*), focal adhesion proteins (*Itga1*, *Itgb4*, and *Itgb5*) and desmosomes (*Dsc2* and *Jup*). The *Cldn6* gene was found to be highly upregulated at all time points after sodium arsenite exposure, however, the statistical significance was observed at 1 h post exposure only. Although at the 1 h time point two gap junction proteins showed upregulation, they were not significantly regulated. At the 1 h time point only, three downregulated genes showed statistical significance. At the 24 h time point, several genes were downregulated; however, only *Itga6* and *Igam* showed statistical significance. At the 4 h time point, gene downregulation patterns were consistent with the 48 h time point. The downregulated genes once again encode tight junction proteins (*Cdh1*, *Cldn7*, *Gjb6*, *Itgb4*, *Ocln*, *Gapdh*, and *Tjp2*), focal adhesion proteins (*Itga5* and *Itgb5*), desmosomes (*Jup*), hemidesmosomes (*Plec*) and gap junction proteins (*Gjb2).* These data collectively reveal that more genes are downregulated during arsenic IV exposure as compared to the arsenic OG exposure (while comparing their respective control groups).

When analyzing mRNA expression of gut-permeability-related genes after the OG, seven genes were significantly upregulated 24 h after arsenite exposure. The data demonstrate that four genes were involved in encoding tight junction proteins (*Cldn1*, *Cldn3*, *Cldn9,* and *Cldn10*). Other significantly upregulated genes encoded gap junction proteins (*Gjb5* and *Gjd2*) and desmosomes (*Dsg4*). After 48 h of sodium arsenite exposure via OG, six genes were significantly regulated. Of these genes, only *Cldn4* was upregulated. Of the remaining genes, two genes encoding tight junction proteins (*Icam2* and *Jam3)* were downregulated. The other three downregulated genes are characterized as encoding focal adhesion (*Itga1* and *Itgal*) and adherens junction proteins (*Notch4*).

**Figure 4:**
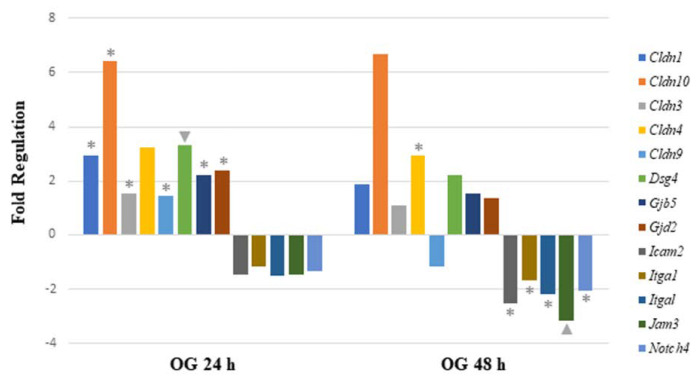
**Comparison of mRNA expression of cell junction pathway genes after 24 h or 48 h oral exposure to sodium arsenite.** Plot of the fold regulation of significantly regulated cell junction genes in ileal tissue of mice exposed to arsenic via oral gavage (OG) for 24 and 48 h. The legend shown below the plot indicates the range of *p*-values (* = p<0.05; ▲ = p<0.01) represented by either marker; *p*-values were calculated based on a Student’s *t*-test of the replicate 2^(−ΔΔCT) values for each gene in the control group and treatment groups.

Comparing the altered gene expression in mice exposed to OG between the 24 and 48 h time points revealed that most of the tight junction genes were highly upregulated at the 24 h time point, specifically claudins and gap junction genes (Figure 4). In contrast, the above-mentioned genes were slightly upregulated at 48 h, however, they were statistically insignificant. Similarly, a few integrin, junctional adhesion and Notch4 receptor encoding genes were significantly downregulated at 48 h, but not at the 24 h time point. Collectively, longer sodium arsenite exposure significantly perturbs the expression of permeability-related genes.

Comparing gene expression between IV and OG exposure after 48 h, only two cell junction genes were commonly modulated (Figure 3 and Figure 4). *Cldn4*, a gene encoding integral membrane protein claudins that are components of epithelial cell tight junctions, was significantly regulated. *Cldn4* was upregulated after 48 h of OG exposure but downregulated after 48 h of IV exposure. *Itga1* was also commonly modulated by arsenic exposure. *Itga1* encodes the α-1 subunit of integrin receptors, which is involved in cell–cell adhesion and collagen binding. Expression of *Itga1* may play a role in inflammation and fibrosis. *Itga1* was downregulated after 48 h of OG as well as IV exposure.

### 2.4. Exposure to Sodium Arsenite via IV Route Does Not Have Significant Impact on the Secretion of Ileal Chemokines/Cytokines

**Figure 5:**
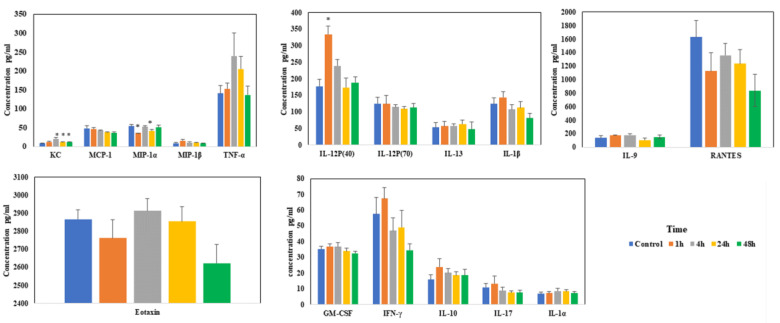
**Effect of arsenic exposure on the gut-associated profile of cytokines and chemokines.** Intestinal ileal mucosal chemokines and cytokine levels were evaluated after mice were exposed to arsenic via IV in the tissue lysate using Bio-Plex mouse cytokine multiplex kits; * above the bar indicated the *p* < 0.05 (Student’s *t*-test) while comparing control group and treatment groups for each cytokine.

Levels of most of the chemokines/cytokines did not change much during sodium arsenite exposure via IV. Among the measured chemokines/cytokines, significant changes were only noticed in the levels of KC, MIP-1α and IL-12P(40) at the early time points (Figure 5). These results of IV exposure clearly contrast with our earlier published results when the exposure was via oral route [22].

### 2.5. Systemic and Local Levels of IgG Isotypes and IgA in Mice Exposed to Arsenite Intravenously Compared to Oral Gavage

**Figure 6:**
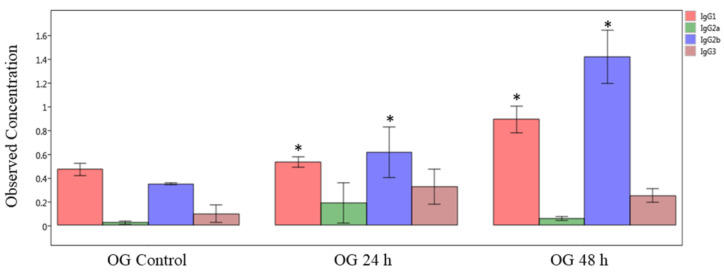
**Comparison of IgG isotype in plasma of sodium-arsenite-treated and control mice.** Plot of observed concentration of IgG isotypes in plasma of control mice and mice exposed orally to sodium arsenic. Significance is represented as a star, where significance indicates p ≤0.05; *p*-values were determined using the Kruskal–Wallis method of statistical analysis.

First, we measured the immunoglobulin isotype levels by multiplex immunoassay of plasma (systemic) samples obtained from mice orally exposed to sodium arsenite (Figure 6). The obtained results show that concentrations of IgG1 and IgG2b significantly increased both at 24 and 48 h after OG arsenite exposure, compared to respective controls (*p* < 0.05). In general, the levels of these two isotypes are more at 48 h compared to 24 h. In contrast, there was no significant difference in plasma Ig levels after IV exposure (data not shown).

**Figure 7:**
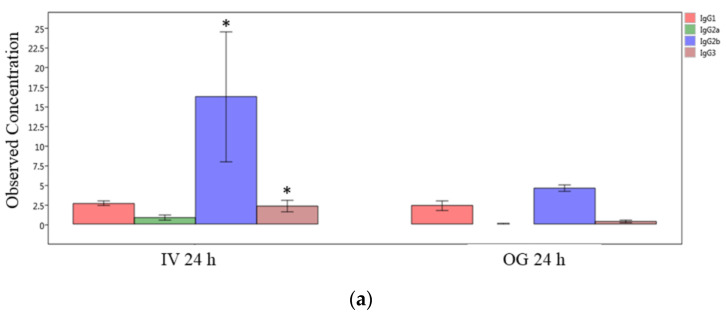

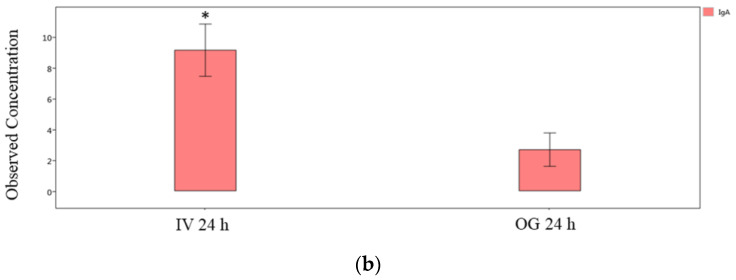
**Comparison of IgG isotypes and IgA in ileal tissue.** Plot of IgG isotypes (top panel; (**a**)) or IgA (lower panel; (**b**)) in ileal tissue of mice after 24 h exposure to sodium arsenite intravenously (IV 24 h) versus orally (OG 24 h). Significance is represented as a star, where significance indicates p ≤0.05; *p*-values were determined using the Mann–Whitney method of statistical analysis.

Finally, we compared the immunoglobulin isotype levels from ileal tissue (local levels) between OG or IV exposed mice at the 24 h and 48 h time points individually. The IgG2b, IgG3 and IgA levels are significantly greater in IV-exposed mice compared to OG mice at 24 h (Figure 7a,b). However, the difference was not statistically significant at 48 h in spite of the higher levels of these immunoglobulins during IV exposure (Supplemental Figure S3).

## 3. Discussion

The results of this study provide evidence that routes of exposure to sodium arsenite modulate gastrointestinal responses (as measured by the gut-mucosa-associated immune response (mRNA expression and immunoglobulin isotyping) and intestinal permeability (mRNA expression)) differently. Our work shows that IV exposure to sodium arsenite led to a transient perturbation of genes regulating host immune response.

Modulation of immune response is evidenced by the significantly differentially regulated genes involved in inflammatory response (*Apcs*, *Crp*, *Cxcl1*, *Cxcl3*, *Il6*, *Slc11a1,* and *Tollip*), cytokine and chemokine secretion (*Cxcl1*, *Cxcl3*, *Ifna9*, *Ifnb1*, *Il12b*, *Il6*, and *Slc11a1*) and apoptosis (*Ifnb*, *Il12b*, *Il6*, *Mpo*, and *Pycard*) between 4 h and 24 h of sodium arsenite exposure. Despite the large number of genes differentially regulated at later time points (40 genes were upregulated 4 h after sodium arsenite exposure and 67 and 66 genes were upregulated 24 h and 48 h, respectively), only a total of four genes showed statistically significant differences between control and sodium-arsenite-treated animals (at 4 h or later), which further support that IV sodium arsenite exposure results in a transient modulation of the expression of genes involved in gut-mucosa-associated immune response. These results contrast with what was observed previously after OG exposure to sodium arsenite [23]. Research from our own laboratory found that OG exposure to sodium arsenite led to a sustained downregulation of genes involved in the NFκB, MEK, JNK/p38, and MyD88-dependent signaling pathways [23].

Observing a decreased concentration of IgG2b and IgA in the ileal tissue of mice exposed to sodium arsenite via OG is consistent with our findings that this route of exposure leads to a greater perturbation of gut-associated immune-response-related genes. In our earlier study, we showed that 24 h after OG exposure to sodium arsenite, there is a significant downregulation of genes involved in Toll-Like Receptor Signaling (*Lbp*, *Ly96*, *Myd88*, *Pik3ca*, *Ripk1*, and *Tlr5*) and NOD-like Receptor Signaling (*Hsp90aa1*, *Naip1*, and *Xiap*), which initiate pro-inflammatory responses [23]. Oral exposure to arsenite led to diminished expression of inflammatory antibody production (IgG2b and IgA) and mRNA for inflammatory genes. By contrast, upregulation of immune response following IV sodium arsenite exposure demonstrated an increased concentration of IgG2b and IgG3 in the ileal tissue and plasma and increased expression of genes encoding pro-inflammatory cytokines and chemokines (*Apcs*, *Crp*, *Cxcl1*, *Cxcl3*, *Ifna9*, *Ifnb1*, *Il12b,* and *Il6)* 24 h post exposure.

Comparison of blood toxicokinetics between OG and IV exposure groups found that the concentration of inorganic arsenic (3+) was much greater in the intestine of mice exposed orally than intravenously [16]. The same study also found that concentrations of erythrocyte-bound arsenic (5+) and arsenic (3+) were nearly equivalent after IV injection but increased nearly 3-fold [arsenic (3+): arsenic (5+)] after OG treatment. These results suggest that sodium arsenite undergoes reductive metabolism to its more toxic species, arsenic (3+), as it passes through the intestine and the liver [15,16]. Via an oral route of exposure, sodium arsenite is absorbed into the gut, where a significant portion undergoes rapid first-pass reductive metabolism into reactive arsenic (3+) and several other metabolites of arsenic by local biotransformation enzymes (bacterial as well as mucosal epithelial cells) [28]. It is then systemically circulated via portal blood flow to the liver for additional reduction. Metabolism of sodium arsenite occurs to a lesser extent after IV injection because sodium arsenite reaches the gut via the mesenteric arteries, at the basal side of intestinal epithelial cells, thus avoiding the biotransformation from gut enzymes and gut microbiota [28]. As a result, there is reduced passage of reactive arsenic species over the gastrointestinal barrier into the intestine following IV exposure.

These proposed mechanisms of sodium arsenite metabolism for different routes of exposure are consistent with what is observed in our gut-associated immune response mRNA expression analyses and immunoglobulin isotyping assays. That is, if the IV route of exposure arsenic is less likely to be transformed into its toxic forms, the host immune response will be less perturbed, as observed (Figure 1). However, when introduced to the body orally, sodium arsenite reaches the intestinal tract directly to undergo metabolism by the intestinal microbes, as well as intestinal epithelial cells. Likely, arsenite and the bio-transformed species in the gastrointestinal tract disrupt the mucosal membrane to cause inflammation. The toxicity of sodium arsenite to the host increased, as it downregulates the activity of key immune-response-related receptors and co-factors. Thus, sodium-arsenite-induced inflammation can persist.

In our experimental model, the pattern of immune response gene expression in mice exposed to sodium arsenite for 24 h represents the innate immune response. Innate immunity is the first line of defense against invasion of the host. Rather than relying on the presentation of antigens, innate immunity rapidly recruits immune cells to a site of infection by the production and release of cytokines and chemokines. Complement systems followed by phagocytosis are also characteristic of the innate immune response, leading to the identification, opsonization and phagocytosis of pathogenic bacteria. This contrasts with the adaptive immune response, which is antigen dependent. Since it involves a lag time between exposure to the antigen and maximal response [29], it is represented by the 48 h sodium arsenite exposure group. Given that the upregulation of immune response genes and pro-inflammatory immunoglobulins is only transient, our results further support that the adverse effects of sodium arsenite are less when exposed to the host intravenously rather than orally. In fact, our prior study represents the pattern of immune response gene expression and transient increase in the abundance of *Clostridium sulfatireducens*, a sulfate-reducing bacterium (SRB), in mice orally exposed to arsenite for initiation of the innate immune response [22]. As the ileal tissue and plasma are from the same set of orally gavaged animals, it could indicate the involvement of these bacteria in the thiolation of arsenic [30]. Rather than relying on the presentation of antigens, innate immunity (gut-associated immune response) rapidly recruits immune cells to the ileal mucosa by the production and release of cytokines and chemokines, as reported for OG [22].

Interestingly, IV sodium arsenite exposure leads to a sustained downregulation of genes involved in encoding tight junction proteins (*Cldn2*, *Cldn4*, *Cldn6, Cldn7*, *F11r*, *Ocln, Tjp1*, *Tjp2,* and *Tjp3)*, focal adhesion proteins (*Itga1*, *Itga5*, *Itga6*, *Itgam*, *Itgb4,* and *Itgb5)*, gap junctions (*Gjb1*, *Gjb2*, *Gjb6* and *Gjc2)* and desmosomes and hemidesmosomes (*Dsc2*, *Jup,* and *Plec*, respectively), all of which are important for regulating intestinal cell permeability and cell–matrix interactions. Although downregulation of cell-junction- and cell-matrix-related genes is observed from 1 h through 48 h after IV exposure, genes were most significantly regulated at 48 h, suggesting that permeability is greatest at this time point (Supplemental Figure S1). The output of IPA analysis is here for the ‘visualization of the analysis patterns’ only [31]. This is consistent for what is observed at 48 h after IV exposure for the RT-PCR analysis of immune-response-related genes. At 1 h after IV sodium arsenite exposure, immune response is depressed, while after 48 h most pro-inflammatory, immune-response-related genes are upregulated. This suggests that an immune response is elicited in response to sodium arsenite exposure. Increased permeability allows arsenic and its metabolites to pass through into the intestinal lumen, where epithelial cell adaptive immunity and inflammatory response are signaled. This prediction is consistent with the results of our immunoglobulin isotyping assays, that the expression of pro-inflammatory immunoglobulins IgG2b and IgA are greatest in both the ileal tissue and plasma samples when sodium arsenite exposure took place via IV rather than OG. Moreover, these immunoglobulins serve as a local protective action in the intestinal lumen [32]. Thus, our findings cumulatively show that IV exposure to sodium arsenite increases gut permeability, which is critical to eliciting an immune response and ultimately detoxification of the xenobiotics from the host.

The opposite is true for OG exposure to sodium arsenite, in which fewer cell-junction- and cell-matrix-related genes were significantly regulated after 24 and 48 h of exposure (Supplemental Figure S2). Yet, we observed a persistent downregulation of immune-response-related genes following OG exposure, suggesting that when exposed orally, sodium arsenite undergoes bioactivation because of decreased epithelial cell permeability. This hypothesis is also conducive with the results of our immunoglobulin isotyping assay, which found that the concentration of IgG1 increased from 24 to 48 h post OG exposure to sodium arsenite, indicating a potent anti-inflammatory effect. In fact, IgG1 production has been shown to correlate with the increased anti-inflammatory activity [33].

A result that appears slightly less consistent is the observation that in plasma samples (IV exposure), the concentration of IgG2b, which is a pro-inflammatory immunoglobulin, also increased between 24 and 48 h, while the mRNA expression of several genes related to intestinal immune response remained depressed. Furthermore, the cytokine/chemokine levels showed very limited perturbation during IV exposure. One possible explanation for this observation is the significant downregulation of the tight junction (*Icam2),* focal adhesion (*Itga1* and *Itgal),* and adherens protein (*Notch4)* genes at 48 h; this might have increased the intestinal permeability, thus allowing for the organic and inorganic species of sodium arsenite to pass through into the intraluminal space, interacting with local immune cells to produce an inflammatory response. Another plausible explanation for this is that the increasing concentration of the IgG2b inflammatory immunoglobulin might be the result of arsenic interaction with the gut microbiota, leading to the immune response within the intestine [20]. A study from our own lab found that after repeated exposure to sodium arsenite in adult mice, there was an abundance of bacteria belonging to the genera *Alistipes*, *Bilophila,* and *Lactobacillus johnsonii*. Bacteria from these genera are characterized by their function in abdominal infection and autoimmune responses. Thus, it may be possible that interaction of arsenic and arsenic-reactive metabolites communicating with commensal bacteria is causing the upregulation of IgG2b in the intestinal lumen despite decreased intestinal permeability. We cannot extrapolate the findings from this study to the effect(s) of long-term arsenic exposure, since the current study only administered a single dose of arsenic. However, these relationships suggest that the intestinal mucosa and the first-pass metabolism should be involved in the risk assessment of xenobiotics and warrant further attention in the future. In conclusion, these results present sufficient evidence in support of the hypothesis that arsenic exposure via OG or IV differentially impacts the expression of genes involved in gut-mucosa-associated immune responses and gastrointestinal permeability.

## 4. Materials and Methods

### 4.1. Animals and Sodium Arsenite Exposure

**Figure 8:**
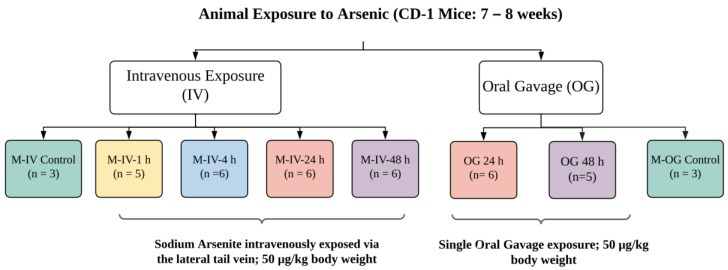
**Schematic diagram representing the design of the animal studies conducted.** A sodium arsenite solution was administered to CD-1 mice intravenously at a concentration of 50 µg/kg body weight via the lateral tail vein. Sacrifices of intravenously exposed mice occurred at 1, 4, 24 or 48 h after exposure. CD-1 mice were also orally dosed with arsenic at the same concentration but were only sacrificed at the 24 or 48 h time points. Ileal tissue and plasma were collected from the sacrificed mice.

All experimental protocols were approved by the Animal Care and Use Committee, which is fully accredited (Food and Drug Administration National Toxicological Research Accreditation #A4310–01) [15,16]. Female CD-1 mice (7 to 8 weeks of age) (for OG) were procured from Charles River, Inc. (Wilmington, MA, USA). To reduce the background level of arsenic, mice were provided a common low-arsenic basal diet (5K96; Test Diets, Purina Mills, Richmond, IN, USA) for at least 10 days prior to arsenic dose administration. Animals for individual experiments were housed in groups. After acclimation, these animals were exposed to a single oral gavage (OG) of sodium arsenite consisting of 0.05 mg/kg body weight (BW), as described earlier [15,16]. In brief, this concentration reflects the intake of arsenic-contaminated water throughout the day that could reach to a daily intake of ~50 μg/kg bw (https://cfpub.epa.gov/ncea/iris_drafts/recordisplay.cfm?deid=309710, accessed on 20 October 2021). At the time of exposure, animals were 9–10 weeks of age with a BW of 30.1 ± 6.7 or 28.5 ± 6.0 g for OG or IV, respectively. These animals were compared to the age-matched animals with vehicle only, as described earlier [15,16]. Animals were sacrificed at 24 or 48 h after oral gavage (Figure 8). For IV injection, adult mice were dosed with sodium arsenite intravenously via the lateral tail vein (5 μL/g BW). Animals were sacrificed 1, 4, 24 and 48 h after the IV injection (Figure 8). The euthanasia in both experimental groups (OG or IV) was administered by CO_2_ asphyxiation as per NIH ARAC guidelines for the euthanasia of rodents using CO2 (https://OACU.OIR.NIH.GOV, accessed on 20 October 2021). A detailed diagram representing the design and number of the animals in each group is presented in Figure 8.

### 4.2. Collection of Ileal Tissue

On the day of sacrifice, all animals were euthanized by exposure to carbon dioxide. Euthanasia was verified by exsanguination conducted prior to initiation of necropsy. The intestine was exposed and flushed with normal saline solution, and a 3 cm section of the terminal ileum was collected in individual cryo-tubes for RNA extraction [22,23]. These cryo-tubes were immediately flash frozen for later use.

### 4.3. Extraction of RNA

The RNA extraction and conversion to cDNA was conducted as described earlier [22,23]. Mouse intestinal tissue was homogenized in TRIzol Reagent (Thermo Fisher, Waltham, MA, USA) using a handheld homogenizer and nuclease-free pestle. For organic extraction, chloroform was added, and the tube was gently vortexed to mix the contents in the tube. This tube was then centrifuged at 10,000× *g* for 20 min at 4 °C, and the top layer of aqueous solution was removed and transferred into a new tube. Equal volumes of isopropanol were added to the samples, which were rested at −20 °C for 2 h. The pellets that precipitated were washed with ice-cold 70% ethanol, air-dried to remove the remaining ethanol and finally resuspended in nuclease-free H_2_O (Thermo Fisher, Waltham, MA, USA).

### 4.4. DNase Treatment and Conversion of RNA to cDNA

The concentration of extracted RNA was measured via Cytation (BioTek, Santa Clara, CA, USA). The 260/280 ratio was above 1.8 for all samples. The known amount of this extracted RNA was treated with TURBO DNase (Thermofisher, Waltham, MA, USA) to remove any genomic DNA contamination. Then, 2.5 μg of DNase-treated RNA samples were mixed with Superscript IV VILO Mastermix (Thermofisher, Waltham, MA, USA) for synthesis of cDNA, as described earlier [34].

### 4.5. mRNA Expression of Gut-Mucosa-Associated Immune Response and Intestinal-Permeability-Related Genes

cDNA samples from the intestinal tissue (OG, 24 or 48 h; IV, 1, 4, 24 or 48 h post exposure) were used for Real-Time Polymerase Chain Reaction (RT-PCR) to detect the transcription levels of 84 genes involved in various aspects of immune status or intestinal permeability. Specifically, PAMM-148Z and PAMM-213Z plates (Qiagen, Germantown, MD, USA) were used to assess genes involved in the immune status or epithelial cell junction pathway, respectively. Please note that the mRNA expression of immune-response-related genes for OG 24 or 48 h was published earlier [23]. cDNA samples were mixed with SYBR Green Master mix (Qiagen), and the samples were aliquoted into PCR array wells. The prepared assays were then run in a Bio-Rad CFX384 Real-Time PCR machine (Bio-Rad, Hercules, CA, USA), using the following conditions: 95 °C for 10 min, followed by 40 cycles of 95 °C for 15 s and 60 °C for 1 min. In addition, a melt curve analysis was performed to verify the purity of each product.

### 4.6. Protein Preparation from the Ileal Tissue for Multiplex Cytokine/Chemokine Levels and Immunoisotyping

Protein from mice ileal tissue was extracted using a gentleMACS dissociator (Miltenyi Biotec, Inc., Auburn, CA, USA), as described earlier [22]. In brief, after the lysate preparation, the resulting cell extract was centrifuged at 60× *g* for 10 min at 4 °C. The clear homogenate was transferred to 2 mL Eppendorf tubes and centrifuged at 4 °C for 15 min at 15,294× *g*, and the resulting suspension was transferred into new 2 mL microtubes and stored at −80 °C until utilized for an immunoglobulin assay. The concentration of protein was measured using Bio-Rad protein assay reagent (Bio-Rad, Hercules, CA, USA). A stock solution of 900 µg/mL was made for multiplex assays. These samples were further diluted for isotyping. For IgA, measurement protein concentration of 30.0 µg/mL was found to be an optimal concentration, whereas for all other IgG isotypes and for IgM, a concentration of 450 µg/mL was found to be optimal. Dilutions were prepared in the assay buffer provided with the immunoassay kit.

### 4.7. Multiplex Chemokine/Cytokine Assay

Ileal-tissue-associated chemokine and cytokine levels were evaluated in the ileal lysate (as prepared above) using a Bioplex mouse cytokine 23-plex panel (Bio-Rad, Hercules, CA, USA) following the manufacturer’s instructions and as previously described using the multiplex Bioplex assay [22].

### 4.8. Plasma Collection

Blood was collected using EDTA tubes (BD Vacutainer from Beckton, Dickinson and Company, Franklin Lakes, NJ, USA), placed on a rocker and centrifuged to collect plasma, as described earlier [15]. All plasma samples were stored frozen at −80 °C until analyzed. Plasma samples of sodium-arsenite-exposed (both IV and OG) mice were prepared according to the Mouse Immunoglobulin Isotyping Magnetic Bead Panel Protocol provided by Millipore (Millipore Sigma, Burlington, MA, USA). Stored plasma samples were first thawed and centrifuged. These samples were then diluted, and immunoglobulin isotype concentrations were measured using the multiplex assay according to the manufacturer’s instructions.

### 4.9. Immunoassay Procedure: Immunoglobulin Isotyping

Immunoglobulin isotyping was achieved by utilizing the Mouse Immunoglobulin Isotyping Magnetic Bead Panel (Millipore Sigma, Burlington, MA, USA). This is a multiplex kit used for the quantification of IgM, IgG1, IgG2a, IgG2b, IgG3, and IgA both from plasma and ileal tissue. Manufacturer’s instructions were followed to measure all immunoglobulin concentrations in plasma samples. For ileal tissue, a 30.0 µg/mL concentration was used to detect IgA and a 57 µg/mL concentration was used to measure IgG isotypes and IgM. For isotyping all biological samples were run in duplicate.

### 4.10. Data Analysis

mRNA gene expression data were analyzed using the Qiagen GeneGlobe Data Analysis Center with a threshold of 40 cycles [35]. Data were normalized using β-actin, β-2 microglobulin, GAPDH and Hsp90ab as housekeeping genes. Results were expressed as fold regulation values of genes at a particular time post exposure as compared to the respective exposure-route control. Fold regulation values from Qiagen Data Analysis Center were used in data representation. A fold change of 1.5 with a *p*-value ≤ 0.05 was considered significant. To gain further insight into the perturbation of genes with sodium arsenite, Ingenuity Pathway Analysis (IPA; v8.0, Qiagen) was used (http://www.ingenuity.com, accessed on 7 September 2020). Fold-change and *p*-value tables obtained from GeneGlobe data analysis were acquired as the input data for IPA. Output of IPA provides status for each gene perturbed in the pathway.

Data from the immunoglobulin isotyping panel were analyzed using the Bio-Plex Manager and Bio-Plex Data Pro software (version 6.2). The concentration of immunoglobulins in plasma and tissue samples was calculated based on a standard curve generated from serially diluted standards according to instructions provided by the vendor. Results were expressed as observed concentrations with respect to the standard. Kruskal–Wallis and Mann–Whitney tests of significance were used to make comparisons between treatment groups and their respective route- and time-matched vehicle controls. Mann–Whitney tests were used when only two treatment groups were compared, whereas Kruskal–Wallis tests were utilized for comparisons between more than two groups. As above, *p*-values ≤ 0.05 were considered as statistically significant. Comparisons were made between exposures routes and treatment periods.

## 5. Conclusions

In conclusion, these results present sufficient evidence in support of the hypothesis that arsenic exposure via OG or IV differentially impacts the expression of genes involved in gut-mucosa-associated immune responses and gastrointestinal permeability. We conclude that IV exposure to sodium arsenite upregulates immune and inflammatory responses, as demonstrated by upregulated mRNA expression of immune response genes and increased concentrations of pro-inflammatory immunoglobulins. Increased epithelial cell permeability was also observed following IV exposure. As a result, the toxicity of xenobiotics, such as arsenic, is reduced via intravenous exposure, as an elicited immune response can lead to toxicant detoxification. This contrasts with OG exposure to sodium arsenite in which inflammatory response is downregulated and anti-inflammatory immunoglobulin concentration increases. Corroborating these results with the decreased epithelial cell permeability, we may conclude that OG exposure of xenobiotics can be more toxic due to toxicant bioactivation. Despite the significance of these findings, we cannot extrapolate the findings from this study to the effect(s) of long-term arsenic exposure since the current study only administered a single dose of arsenic. However, these relationships suggest that the intestinal mucosa and the first-pass metabolism should be involved in the risk assessment of xenobiotics and warrant much attention in the future.

## Figures and Tables

**Table 1:** Altered mRNA expression of immune-response-related genes.

	IV 1 h	IV 4 h	IV 24 h	IV 48 h
Upregulated	39	40	67	66
Downregulated	45	44	17	18
Statistically Significant	17	1	1	2

Table 1 provides number of immune-response-related genes differentially expressed vs. time- and route-matched controls.

**Table 2:** Altered mRNA expression of cell–cell-junction-related genes.

	IV 1 h	IV 4 h	IV 24 h	IV 48 h	OG 24 h	OG 48 h
Upregulated	36	23	47	60	59	50
Downregulated	48	61	37	24	25	34
Statistically Significant	4	11	2	18	7	6

Table 2 represents the number of cell–cell-junction-related genes differentially expressed vs. time- and route-matched controls. Please note that OG immune-response-related gene data were previously published by our group [23] (https://doi.org/10.1016/j.fct.2019.110597).

## Data Availability

Data will be available as per the US-FDA data sharing rules.

## Supplementary Materials

The following supporting information can be downloaded at: https://www.mdpi.com/article/10.3390/ijms24076352/s1.

## Abbreviations

Ig	Immunoglobulin
IV	Intravenous
OG	Oral gavage
IPA	Ingenuity Pathway Analysis

## References

[1] National Institute of Environmental Health Sciences, U.S. Department of Health and Human Services (2020). Arsenic. www.niehs.nih.gov/health/topics/agents/arsenic/index.cfm.

[2] (2017). www.who.int/ceh/capacity/heavy_metals.pdf.

[3] Barchowsky A., Klei L.R., Dudek E.J., Swartz H.M., James P.E. (1999). Stimulation of reactive oxygen, but not reactive nitrogen species, in vascular endothelial cells exposed to low levels of arsenite. Free. Radic. Biol. Med..

[4] Ratnaike R.N. (2003). Acute and chronic arsenic toxicity. Postgrad. Med. J..

[5] Das N., Paul S., Chatterjee D., Banerjee N., Majumder N.S., Sarma N., Sau T.J., Basu S., Banerjee S., Majumder P. (2012). Arsenic exposure through drinking water increases the risk of liver and cardiovascular diseases in the population of West Bengal, India. BMC Public Health.

[6] Ruan Y., Fang X., Guo T., Liu Y., Hu Y., Wang X., Hu Y., Gao L., Li Y., Pi J. (2022). Metabolic reprogramming in the arsenic carcinogenesis. Ecotoxicol. Environ. Saf..

[7] Ozturk M., Metin M., Altay V., Bhat R.A., Ejaz M., Gul A., Unal B.T., Hasanuzzaman M., Nibir L., Nahar K. (2022). Arsenic and Human Health: Genotoxicity, Epigenomic Effects, and Cancer Signaling. Biol. Trace Elem. Res..

[8] Kile M.L., Houseman E.A., Baccarelli A., Quamruzzaman Q., Rahman M., Mostofa G., Cardenas A., Wright R., Christiani D.C. (2014). Effect of prenatal arsenic exposure on DNA methylation and leukocyte subpopulations in cord blood. Epigenetics.

[9] Jin Y., Xi S., Li X., Lu C., Li G., Xu Y., Qu C., Niu Y., Sun G. (2006). Arsenic speciation transported through the placenta from mother mice to their newborn pups. Environ. Res..

[10] Ettinger A.S., Zota A.R., Amarasiriwardena C.J., Hopkins M.R., Schwartz J., Hu H., Wright R.O. (2009). Maternal arsenic exposure and impaired glucose tolerance during pregnancy. Environ. Health Perspect..

[11] Devesa V., Adair B.M., Liu J., Waalkes M.P., Diwan B.A., Styblo M., Thomas D.J. (2006). Arsenicals in maternal and fetal mouse tissues after gestational exposure to arsenite. Toxicology.

[12] Bozack A.K., Cardenas A., Geldhof J., Quamruzzaman Q., Rahman M., Mostofa G., Christiani D.C., Kile M.L. (2020). Cord blood DNA methylation of DNMT3A mediates the association between in utero arsenic exposure and birth outcomes: Results from a prospective birth cohort in Bangladesh. Environ. Res..

[13] Wang Z., Zhou J., Lu X., Gong Z., Le X.C. (2003). Arsenic Speciation in Urine from Acute Promyelocytic Leukemia Patients undergoing Arsenic Trioxide Treatment. Chem. Res. Toxicol..

[14] Chi L., Bian X., Gao B., Tu P., Ru H., Lu K. (2017). The Effects of an Environmentally Relevant Level of Arsenic on the Gut Microbiome and Its Functional Metagenome. Toxicol. Sci..

[15] Twaddle N.C., Vanlandingham M., Beland F.A., Doerge D.R. (2019). Metabolism and disposition of arsenic species from controlled dosing with dimethylarsinic acid (DMA(V)) in adult female CD-1 mice. V. Toxicokinetic studies following oral and intravenous administration. Food Chem. Toxicol..

[16] Twaddle N.C., Vanlandingham M., Fisher J.W., Doerge D.R. (2018). Metabolism and disposition of arsenic species from controlled dosing with sodium arsenite in adult female CD-1 mice. III. Toxicokinetic studies following oral and intravenous administration. Food Chem. Toxicol..

[17] Doherty M.M., Pang K.S. (1997). First-Pass Effect: Significance of the Intestine for Absorption and Metabolism. Drug Chem. Toxicol..

[18] Sharma S., Kaul D., Singh D. (2015). Arsenic toxi-RNomics has the ability to tailor the host immune response. Exp. Mol. Pathol..

[19] Dheer R., Patterson J., Dudash M., Stachler E.N., Bibby K.J., Stolz D.B., Shiva S., Wang Z., Hazen S.L., Barchowsky A. (2015). Arsenic induces structural and compositional colonic microbiome change and promotes host nitrogen and amino acid metabolism. Toxicol. Appl. Pharmacol..

[20] Lu K., Abo R.P., Schlieper K.A., Graffam M.E., Levine S.S., Wishnok J.S., Swenberg J.A., Tannenbaum S.R., Fox J.G. (2014). Arsenic Exposure Perturbs the Gut Microbiome and Its Metabolic Profile in Mice: An Integrated Metagenomics and Metabolomics Analysis. Environ. Health Perspect..

[21] Tikka C., Manthari R.K., Ommati M.M., Niu R., Sun Z., Zhang J., Wang J. (2020). Immune disruption occurs through altered gut microbiome and NOD2 in arsenic induced mice: Correlation with colon cancer markers. Chemosphere.

[22] Gokulan K., Arnold M.G., Jensen J., Vanlandingham M., Twaddle N.C., Doerge D.R., Cerniglia C.E., Khare S. (2018). Exposure to Arsenite in CD-1 Mice during Juvenile and Adult Stages: Effects on Intestinal Microbiota and Gut-Associated Immune Status. mBio.

[23] Arnold M.G., Gokulan K., Doerge D.R., Vanlandingham M., Cerniglia C.E., Khare S. (2019). A single or short time repeated arsenic oral exposure in mice impacts mRNA expression for signaling and immunity related genes in the gut. Food Chem. Toxicol..

[24] Chiocchetti G.M., Vélez D., Devesa V. (2019). Inorganic arsenic causes intestinal barrier disruption. Metallomics.

[25] Seo K., Seo J., Yeun J., Choi H., Kim Y.-I., Chang S.-Y. (2021). The role of mucosal barriers in human gut health. Arch. Pharmacal Res..

[26] Oshima T., Miwa H. (2016). Gastrointestinal mucosal barrier function and diseases. J. Gastroenterol..

[27] Kile M.L., Baccarelli A., Hoffman E., Tarantini L., Quamruzzaman Q., Rahman M., Mahiuddin G., Mostofa G., Hsueh Y.-M., Wright R. (2012). Prenatal Arsenic Exposure and DNA Methylation in Maternal and Umbilical Cord Blood Leukocytes. Environ. Health Perspect..

[28] Hinrichsen S., Geist F., Planer-Friedrich B. (2015). Inorganic and Methylated Thioarsenates Pass the Gastrointestinal Barrier. Chem. Res. Toxicol..

[29] Marshall J.S., Warrington R., Watson W., Kim H.L. (2018). An introduction to immunology and immunopathology. Allergy Asthma Clin. Immunol..

[30] Rubin S.S.C.D.C., Alava P., Zekker I., Laing G.D., de Wiele T.V. (2014). Arsenic thiolation and the role of sulfate-reducing bacteria from the human intestinal tract. Environ. Health Perspect..

[31] Merico D., Gfeller D., Bader G.D. (2009). How to visually interpret biological data using networks. Nat. Biotechnol..

[32] Brown W.R. (1978). Relationships between immunoglobulins and the intestinal epithelium. Gastroenterology.

[33] Mosmann T.R., Coffman R.L. (1989). TH1 and TH2 cells: Different patterns of lymphokine secretion lead to different functional properties. Annu. Rev. Immunol..

[34] Gokulan K., Williams K., Orr S., Khare S. (2020). Human Intestinal Tissue Explant Exposure to Silver Nanoparticles Reveals Sex Dependent Alterations in Inflammatory Responses and Epithelial Cell Permeability. Int. J. Mol. Sci..

[35] https://geneglobe.qiagen.com/us/analyze/.

