# Supplementary material for: Route of Arsenic Exposure Differentially Impacts the Expression of Genes Involved in Gut-Mucosa-Associated Immune Responses and Gastrointestinal Permeability
Source: Int J Mol Sci. 2023 Mar 28;24(7):6352. doi: 10.3390/ijms24076352 (PMC10094558; doi:10.3390/ijms24076352)
Supplement: Supplementary file 1 [file ijms-24-06352-s001.zip › ijms-2250078-supplementary.pdf]

Supplemental Information

Route of Arsenic Exposure Differentially Impacts the Expression of Genes Involved in Gut-Mucosa-Associated Immune Responses and Gastrointestinal Permeability

Kuppan Gokulan[1, †] Aakriti Mathur[1, †], Amit Kumar[1], Michelle M. Vanlandingham[2], and Sangeeta Khare[1*]

[1]Division of Microbiology, National Center for Toxicological Research, US Food and Drug Administration, 3900 NCTR Rd, Jefferson, AR 72079

[2]Division of Biochemical Toxicology, National Center for Toxicological Research, US Food and Drug Administration, 3900 NCTR Rd, Jefferson, AR 72079

[†] ***Equal contribution***

[*]**Corresponding Author:**

Division of Microbiology

National Center for Toxicological Research, US FDA

3900 NCTR Rd., Jefferson, AR 72079

Phone: 870-543-7519; sangeeta.khare@fda.hhs.gov

1 h 4 h 48 h

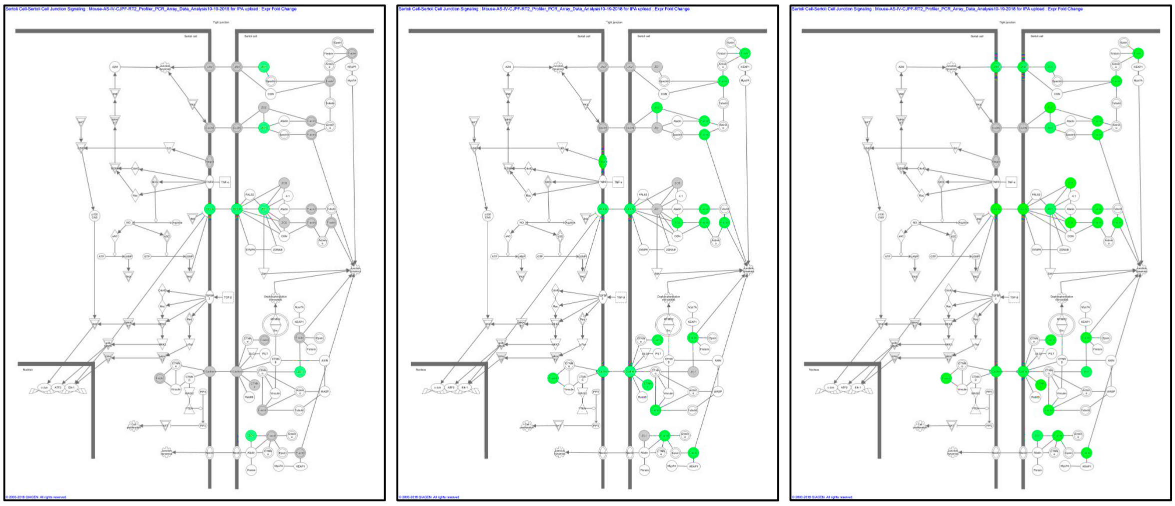

Supplemental Figure S1. **Ingenuity Pathway Analysis of differential expressed genes in the cell-cell junction pathway during sodium arsenite IV exposure.** This schematic diagram shows the interaction and expression of genes involved in the cell-cell junction pathway in the intestinal mucosa during sodium arsenite exposure. Sodium arsenite IV exposure initiate a cascade of reaction for the perturbation of mRNA expression of cell junction related genes as early as 1 h post exposure, with a maximum perturbation at 48 h post exposure. A fold change 1.5 with a p-value ≤ 0.05 were considered significant. Green circles represent down regulation, red circles represent upregulation, and gray circles represent no perturbation.

OG 24 h **OG 48 h**

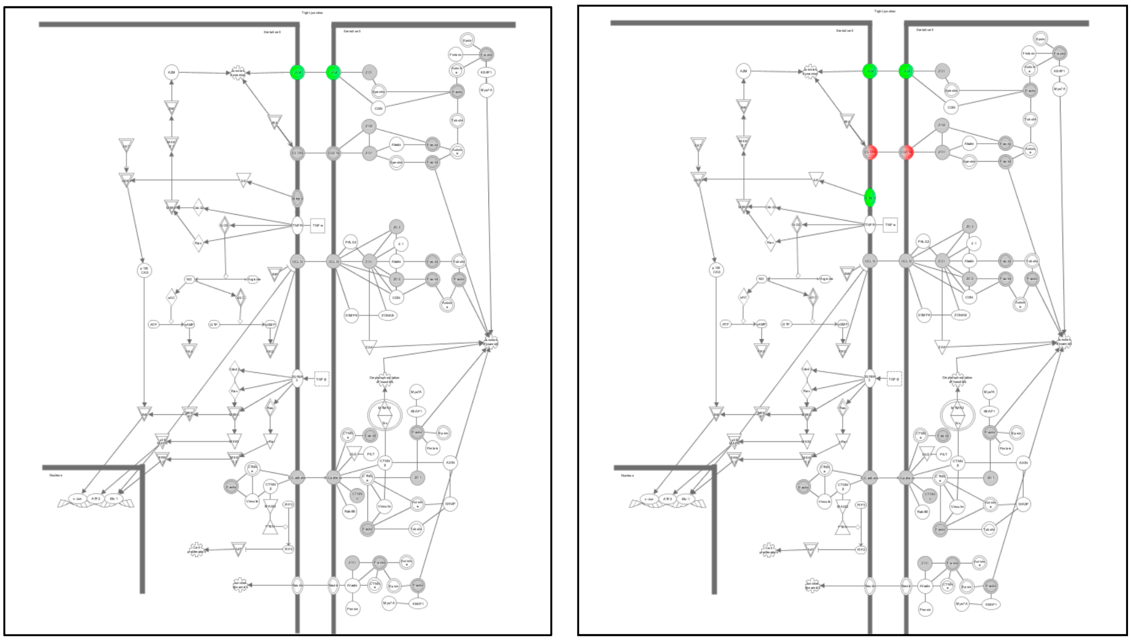

Supplemental Figure S2. Ingenuity pathway analysis of genes in the cell-cell junction pathway during sodium arsenite oral gavage. This schematic diagram shows the interaction and expression of genes involved in the cell-cell junction pathway in the intestinal mucosa during sodium arsenite oral exposure. A fold change 1.5 with a p-value ≤ 0.05 were considered significant. Sodium arsenite oral exposure had very limited perturbation of mRNA expression of cell junction related genes. Green circles represent down regulation, red circles represent upregulation, and gray circles represent no perturbation.

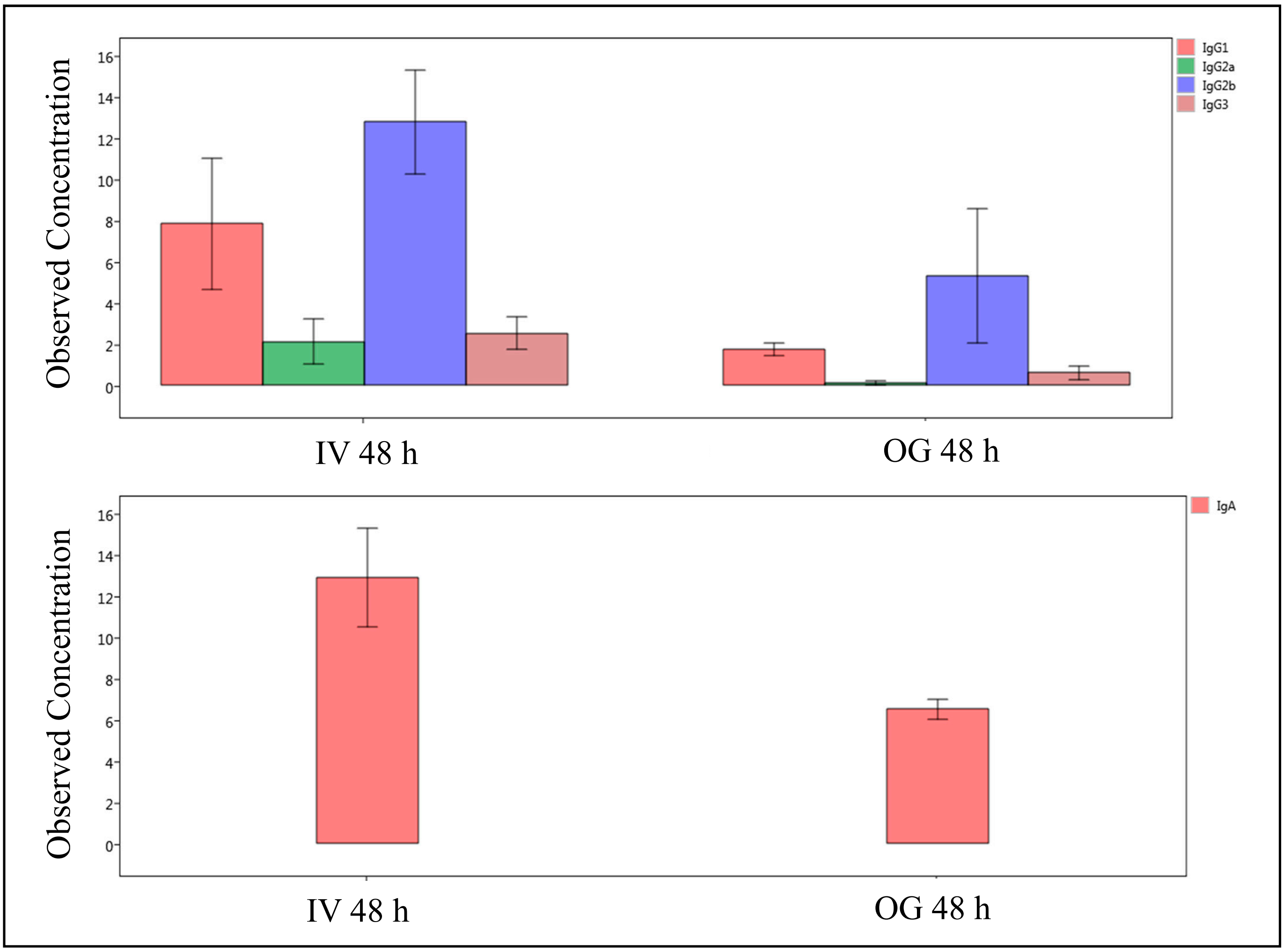

Supplemental Figure S3: **Comparison of IgG Isotypes and IgA in Ileal Tissue.** Plot of IgG isotypes (Top panel; a) or IgA (lower panel; b) in ileal tissue of mice after 48 h exposure to sodium arsenite intravenously (IV 48 h) versus orally (OG 48 h). Significance is represented as a star, where significance indicates $p \leq 0.05$. P-values were determined using the Mann-Whitney method of statistical analysis.
